# How Discharge Instructions Can Change a Life

**DOI:** 10.3928/24748307-20180202-01

**Published:** 2018-03-09

**Authors:** Joseph M. Geskey

Eight years ago my father was being released home from the large academic hospital where I worked, and I was able to be present for his discharge instructions. I was not wearing my white coat or hospital badge and, therefore, was treated as a layperson in this specialized unit because I didn't visit it routinely in my clinical practice. **Table 1** is an approximation of the discharge summary my father received. It should be noted that my father was actually taking 25 mcg of fentanyl (not the 125-mcg dose he was erroneously going to be discharged on) to dull the pain and burning he felt in his mouth and throat from the chemotherapy and radiation he was receiving as part of his treatment regimen for stage III head and neck cancer. The combination of pain and loss of appetite led to the placement of a gastrostomy tube so that he could receive adequate nutrition, hydration, and prescription medications. There were several duplications of medications in different formulations, frequencies, and routes of administration, such as ciprofloxacin, prochlorperazine, and a prescription mouthwash. Also, the medical abbreviation “PO” and the more patient-friendly term “orally” were used interchangeably. When my father received these instructions he was asked if he had any questions, and as anyone who has been present when discharge instructions are given to a friend or family member can probably attest, he quickly answered, “No.”

Although my father has only a high-school education, he does not have low health literacy at baseline, and when he felt better, I showed him the discharge summary and asked him why he didn't mention the obvious discrepancies. He said, “Joe, I barely knew my name and birth date during this time.” To my institution's credit, they supported me telling my father's story throughout the organization, but I couldn't say that there were any system-level improvements that would prevent this from occurring to another patient.

Serendipitously, while my father was hospitalized, my institution was participating in a national study that was investigating whether the implementation of a set of “best practices” for hospital discharge care transitions would reduce 30-day readmissions (Hansen et al., 2013). One of the key interventions was use of the Teach-Back method, which asks patients to state in their own words what was just taught to them by a health care provider. When I adopted this in my clinical practice, I estimated that more than one-third of patients and/or their caregivers could not immediately repeat what I just explained or demonstrated to. I certainly believe Teach-Back would have been helpful during my father's discharge, both to highlight his lack of understanding and the inconsistencies that were present in his discharge instructions. These two revelatory experiences highlighted the incongruity between how and what we expect from our patients regarding self-management skills and the reality of their ability to effectively execute new skills during periods of extreme vulnerability, which can lead to functional impairments in health literacy.

The biology of aging, the impact of illness, and baseline level of education are all important variables that affect a patient's memory, executive function, and processing speed, and they need to be considered in effectively communicating information about illness, treatment options, and self-management skills (Nutbeam, 2000). The inability to successfully execute these skills could have potentially life-limiting implications for patients, and yet our health care systems' educational efforts, whether in the inpatient or outpatient setting, don't receive the same degree of attention as other aspects of health care (Cawthon, Mion, Willens, Roumie, & Kripalani, 2014). Research by Liang and Brach (2017) almost a decade after my father's experience showed that fewer than one-third of patients are asked to Teach-Back instructions from their providers, and only 30% reported that the instructions they received from providers were easy to understand despite expert recommendations to adopt health literacy universal precautions that make health information easier to comprehend.

The absence of my white coat on the day of my father's discharge allowed me to see how difficult it is to effectively assess understanding when people are ill, in pain, just want to go home, and may have limited social support, low formal education, and limited economic resources. My father's experience, combined with my own clinical experience, inspired me to devote my clinical and administrative time in my current organization to create a customized educational approach based on patients' health literacy and activation levels, and more broadly to advocate for the adoption of health literacy universal precautions and more fully develop features of a health literate organization. These interventions can make it easier for health care personnel, caregivers, and patients to effectively partner to achieve success in care transition settings.

## Figures and Tables

**Table 1:** Discharge Summary

**Medication**	**Dose**	**Special Instructions**
Maalox^a^ (lidocaine-diphenhydramine) mouthwash	See instructions	15-mL swish and spit every 6 hours as needed for mouth and throat pain
Maalox^a^ (lidocaine-diphenhydramine) (Magic Swizzle)	See instructions	15-mL swish and spit every 6 hours as needed for mouth and throat pain
Albuterol (Proventil^b^ 0.083% for nebulization)	Inhalation every 6 hours	
Amoxicillin-clavulanate (amoxicillin 400 mg; clavulanate 57 mg/5 mL oral suspension)	Take 10 mL by mouth or PEG twice daily for 10 days	
Aprepitant (aprepitant 80–125 mg kit)	Take as directed	
Ciprofloxacin (Cipro)^c^	See instructions	5 mL by mouth twice daily for 10 days (please take orally and do not put through PEG tube)
Ciprofloxacin (ciprofloxacin 500 mg/5 mL oral suspension)	Take 5 mL by mouth 2 times a day for 10 days	
Ciprofloxacin (ciprofloxacin 500-mg tablet)	Take 1 tablet by mouth every day	
Dexamethasone (dexamethasone 4-mg tablet)	Take 1 table by mouth every 12 hours for 4 days after chemotherapy	
Docusate (Colace^d^)	100 mg by mouth 2 times daily	Melt capsule in 20 mL of warm water and give through gastrostomy tube
Emollients (topical) (Biafine^e^)	1 application topically 3 times daily	
Fentanyl	See instructions	100-mcg patch every 3 days
Fentanyl (fentanyl 25 mcg/hour patch)	Apply 1 patch transdermally every 72 hours	
Fluconazole (Diflucan^f^)	See instructions	2.5 mL by mouth daily (to end on 3/31/09)
Fluticasone-salmeterol (Advair Diskus^a^ 100–50 mcg)	1 puff inhalation 2 times daily	
Ondansetron (Zofran^a^)	See instructions	8 mg by mouth twice daily for 3 days
Prochlorperazine (Compazine^a^)	See instructions	10 mg by mouth every 4 hours as needed for nausea
Prochlorperazine (10-mg tablet)	Take 1 tablet by mouth every 6 hours as needed	
Ranitidine (Zantac^g^)	300 mg once daily	
Scopolamine	See instructions as needed for secretions	1 patch every 3 days as needed for secretions

Note. PEG, percutaneous endoscopic gastrostomy.

a GlaxoSmithKline, Middlesex, UK.

b Merck and Co., Kenilworth, NJ.

c Bayer USA, Pittsburgh, PA.

d Purdue Products, Stamford, CT.

e Laboratoire Medix, Houdan, France.

f Pfizer, New York, NY.

g Chattem Inc., Chattanooga, TN.
